# Nurses' experiences working with nursing students in a hospital: a phenomenological enquiry

**DOI:** 10.1590/1518-8345.1242.2788

**Published:** 2016-07-25

**Authors:** Yolanda Raquel Lapeña-Moñux, Luis Cibanal-Juan, Mª Isabel Orts-Cortés, Mª Loreto Maciá-Soler, Domingo Palacios-Ceña

**Affiliations:** 1 Assistant Professor, Department of Nursing, Universidad Jaume I, Spain.; 2 Full Professor, Department of Nursing, Universidad de Alicante, Spain.; 3 Full Professor, Department of Nursing, Universidad Jaume I, Spain.; 4 Full Professor, Department of Nursing, Universidad de Alicante, Spain.; 5 Assistant Professor, Department of Health Science, Universidad Rey Juan Carlos. Spain.

**Keywords:** Education Nursing, Hospitals, Students Nursing, Qualitative Research

## Abstract

**Objective:**

this paper explores the experiences of registered nurses working with Spanish nursing students within the hospital.

**Methods:**

a qualitative phenomenological approach was followed. Purposeful sampling was employed. Twenty-one registered nurses, from a public hospital located in Spain, were included in the study. Data were collected by means of unstructured and semi-structured interviews and were analysed using Giorgi's proposal. The Consolidated Criteria for Reporting Qualitative Research were followed.

**Results:**

three main themes described the experience of registered nurses: "The nurse's relationship with nursing students"; most nurses emphasized the importance of the first contact with students and they considered students' attitude to be key. "Defining the role of the student in clinical practice"; it is necessary to unify the nurse's role and interventions to avoid misleading students and establish priorities in clinical practice. "Building bridges between clinical settings and the University"; the need to establish a common ground and connection between the university and hospital clinical settings was emphasized. Nurses felt that the training program should also be designed by the clinical settings themselves.

**Conclusions:**

understanding the meaning of nursing students with registered nurses might gain a deeper insight into their expectations.

## Introduction

Clinical practice is an important part of the nursing curriculum, in which students apply the knowledge acquired at university^1^. Clinical practice requires students to adapt to a complex and changing environment in which they must interact with multiple professionals^2^. During this process, professional nurses are essential for the appropriate training and adaptation of the students. They teach, guide and monitor, as well as facilitate integration of trainees into the clinical setting^3^ ^-^ ^6^. 

In Spain, nursing programs follow the European guidelines of the European Higher Education Area (EHEA)^7^. The EHEA aims to increase compatibility among national higher education systems (university level courses). The curriculum of nursing studies comprises two basic components: a) theoretical content; and b) training in competencies, abilities and technical skills. These components are covered both at the university as well as within clinical settings, such as hospitals. The bachelor's degree in nursing includes 32.5% of credits intended for training provided in clinical settings^8^. In Spain, universities develop nursing curricula, however, nursing students acquire their main skills during clinical placements. University nursing departments are in charge of selecting hospital professionals responsible for monitoring students. These professionals, referred to as associate professors, are the key communication link between clinical settings and the university. During their clinical placements, students are welcomed by the nursing unit team, and each student is assigned a reference nurse. 

In this study, the nurses who work in clinical settings are referred to as registered nurses (RN). These nurses have no contractual relationship with the university and their purpose is to teach, guide and facilitate students' integration into the clinical environment. Likewise, the term 'associate professor' will be used for reference nurses or nurse managers who are hired by the university to monitor a group of students at the hospital. Clinical placements provide opportunities for professional socialization by allowing nursing students to experience how staff nurses interact, feel, and think, as well as what they value^1^. The degree of learning, skill development and confidence of students is influenced by their relation with nurses^4^ ^-^ ^6^ ^,^ ^9^ ^-^ ^11^. In clinical practice, the nurse is said to assume different roles in order to facilitate students' learning, such as a stranger, a resource person, a teacher and a leader^3^. Previous studies show how nurses' attitudes and behaviour towards students can vary^3^ ^-^ ^6^, influencing their integration into the clinical practice environment^2^ ^,^ ^6^ ^,^ ^9^ ^-^ ^10^. The aim of this study was to explore the registered nurses' experiences of working with Spanish nursing students.

## Methods

A phenomenological qualitative study was conducted using Giorgi's method of analysis^12^. Qualitative studies are used to achieve a deeper understanding of, and find explanations for people's behaviour under specific circumstances, such as disease^13^. The main characteristic of this qualitative methodology is that the researcher is intimately involved in data collection and analysis; data collection requires the researcher to interact with the study participants and their social context^13^. 

In the field of qualitative studies, phenomenology attempts to understand how individuals construct their world view on the basis of the meanings used by them, in other words it looks through a window into other people's experiences^14^. The aim of phenomenological studies is to identify the essence of living this experience, the lived experienced is the subjective reflection made by subjects in situations or events in a specific geographical, social and cultural environment. This experience always has a meaning for the person who lived it^14^. Qualitative phenomenological studies therefore use first-person narratives from the patients themselves as data source. 

A 2-phase sampling strategy was adopted. The first phase involved purposeful sampling to gather information from registered nurses. The second phase involved theoretical, or in-depth, sampling in order to gain a deeper understanding of specific aspects of the information obtained during the first phase. Inclusion criteria consisted of RN from a hospital in Soria (Spain), whose length of duty in the hospital was greater than one year. Nurses were not excluded on the basis of the kind of units where they were working. Twenty-one nurses with a mean age of 46 were selected. None of the participating nurses withdrew from the study. 

Researchers made an initial contact with the nurses through the Nurse Manager in each unit. During the initial face-to-face contact, researchers explained to the nurses the purpose and design of the study. A 2-week period was then allowed for nurses to decide whether or not they wished to participate. During the second face-to-face contact, they were asked to provide both informed consent and permission to tape the interviews if they wished to participate in the study. 

A university hospital was used to obtain the data. This was a 250-bed facility, in which the human resources were distributed as follows: 15.88% medical staff; 47.83% nursing staff; and 36.29% management and services staff. The nursing department employed 255 nurses, and there were a total of 70 students assigned to this department. 

Data were collected over a period of one year, from April 2010 until March 2011. The first phase consisted of unstructured interviews^13^, beginning with the following question: "What is your experience with nursing students in the hospital?" The aim was to look for emerging themes and topics that could be further expanded upon, during the second phase of the study. The second phase consisted of semi-structured interviews based on an interview guide and aimed at eliciting further information regarding specific themes and topics of interest which had emerged from the first round of interviews. 

**Figure 1:**
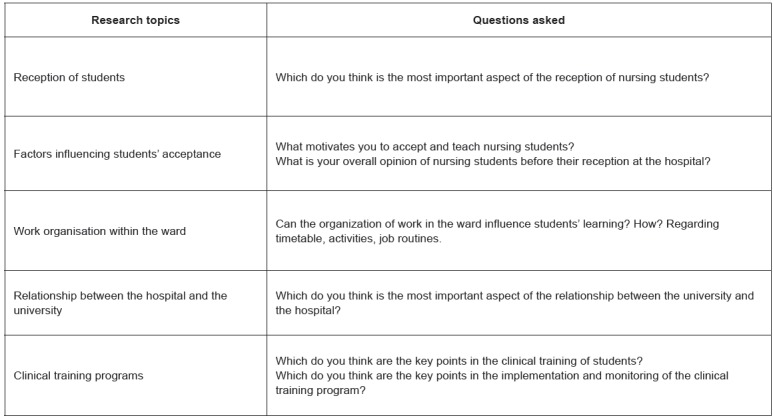
Questions guide for the semi-structured interview. Soria, Castilla León, España, 2012

The interview guide was developed after reviewing the nurses' accounts obtained during the purposeful sampling and following a literature review. It consisted of direct, yet open questions aimed at encouraging nurses to share their experiences. All interviews were taped and transcribed verbatim. Personal documents provided by nurses and researcher field notes were collected during both stages. All the RN included in the study were asked to voluntarily provide any personal documents, such as diaries or letters, where they could describe and explain their experience with the nursing students and their clinical learning process in the hospital. These documents were collated by the researchers. 

A total of 26 interviews involving 21 nurses were conducted: 16 were unstructured interviews (phase 1), and 10 were semi-structured interviews (phase 2). The interviews produced recordings totalling 1988.78 minutes (33.14 hours). One personal letter and five diary entries were collected from the nurses, together with researcher field notes. All interviews were conducted at the hospital.

Full literal transcriptions of each of the interviews, researcher field notes and nurse's documents were produced. Texts were collated to allow qualitative analysis to be performed. 

The method of analysis must be consistent with the type of qualitative design chosen^13^. Giorgi's proposal was selected in order to analyze the experiences of RNs^12^. The Giorgi data analysis proposal allows researchers to capture the essence of the experience through themes as they arise from each subject's story. The transcribed texts were analyzed from a descriptive point of view (words, phrases, metaphors taken from the text) and then interpreted. This material reduction process is intended to extract the essence, or the common thread underlying the experience lived to identify the key contents or *topics* of this experience^12^. An initial analysis aimed at collecting common material, data and information, also known as meaning units. This was performed to construct the emerging topics which describe the experience of RNs working with Spanish nursing students. The second analysis involved grouping the meaning units into common meaning groups and then establishing the main topics. Construction of the meaning units was accomplished using extracts form the passages of text transcribed from the RN's comments. Finally, all the transformed meaning units were synthesized into a consistent statement regarding the subject's experience^12^.

Guidelines for conducting qualitative studies established by the Consolidated Criteria for Reporting Qualitative Research^15^ were followed. The data reliability method consisted of: a) cross-triangulation by the researcher, which included session planning where the cases analysed by each team member were presented in order to reach a consensus; b) audit of the material obtained from 10 randomly selected cases by an external researcher^16^; and c) nurse verification. The nurse's verification was carried out in two steps: post-interview and post-analysis. 

This study was approved by the Clinical Research Ethics Committee of the Santa Barbara Hospital, and was conducted according to accepted national and international standards in accordance with the Declaration of Helsinki in 1995, as revised in Edinburgh 2000^17^.

## Results

Table 1 shows details of the socio-demographic data for the 21 nurses included in the study.

**Table 1:** Sociodemographic data of nurses participants. Soria, Castilla León, España, 2012

Sociodemographic category	Data
Gender	Male: 14,29 % (n=3) Female: 85,71% (n=18)
Average age of nurses	46; (SD^*^ +/- 7.48)
Department/ward	Medical unit. 33.33% (n=7) Surgery unit (orthopaedic, urology, general and digestive surgery). 33.33% (n=7) Haemodialysis unit. 4.76% (n=1) Critical care unit. 9.52%(n=2) Ambulatory care department. 9.52%(n=2) Emergency unit. 9.52%(n=2)
Average years of nurses professional experience	23.33;(SD^*^ +/-7.94)
Average years of work in department/ward	10.52; (SD^*^ +/-10.01)

*SD; Standard Deviation

The themes representing the nurses' experiences of working with the nursing students and the clinical learning process described in the hospital were extracted from the interviews. Three specific themes emerged from the analysed material: a) the nurse's relationships with nursing students; b) defining the role of the student in clinical practice; and c) building bridges between clinical settings and the university. What follows are quotations taken directly from the interviews, diaries and personal letters regarding the 3 emerging themes.

### The nurse's relationships with nursing students

This theme refers to all the elements that facilitate and/or hinder an optimal contact between the student and the nurse. Most nurses emphasized the importance of the first contact with students. It is a time for students to be reassured and guided: *The first contact is critical for the student [...] It is when they want to be liked, to avoid problems during practice. You must reassure them and show them that your relationship will be based on their developing their skills, knowledge and attitude as a nurse, not on being liked [...]* (N5)

Most nurses considered students' attitude to be key. The relationships established with students varied depending on their being considered unmotivated or motivated. Nurses considered motivated students as those showing commitment and hard work from the first day. Students should not wait for the nurse to tell them what they must do: *Some students are not willing to work, to learn. They start making demands and you think: you don't know how to do anything[...]*(N20)

Some accounts described cases of nurses who did not want to participate in student training. At times, the nurse's length of experience was perceived as an obstacle: *People who have been working for a long time and who are not so used to changes seem to find it difficult to teach new things.*(N7)

Other factors that influenced the acceptance of students stemmed from negative experiences at the Nursing School. The lack of rewards for teaching nursing students and the lack of recognition of the nurse's role also influenced the acceptance of new students. Some nurses believed that the training for both professionals and students had not been properly organized by the Nursing School: *There were workmates who refused to teach them because they obtained no reward. When the university decided that particular individuals were to carry out the monitoring task and be paid, many nurses decided not to collaborate [...]*(N15).

### Defining the role of the student in clinical practice

Many nurses spoke of how students must act and what they must do in a clinical setting. Likewise, it is necessary to unify the nurse's role and interventions to avoid misleading students and establish priorities in clinical practice. Most accounts described how students do not seem to know what the nursing profession consists of. This lack of awareness was experienced by nurses as a lack of responsibility on the part of students: *Treatment plans are important to me, however seeing, touching and talking to the patient is even more important. Sometimes I think they don't do so because we don't emphasize the importance of being with the person. We should first define our role as caregivers.*(N8).

Some nurses spoke of the need to establish priorities in clinical practice training. Thus, students would know how to proceed in clinical practice, avoiding student-nurse conflicts: *If they were told what to do, we would avoid many frictions and bad answers. We wouldn't have that feeling of repeating the same things day after day, and we would know exactly what they should be told and taught.*(N4).

Most nurses emphasized that learning priorities have changed, and that students are guided towards a technology demanding care, compared to basic care: *Many students believe that their role is to cling to the doctor and manipulate devices. We constantly tell them that their duty is to remain close to the patient and their family, not to handle devices.*(N8).

Likewise, nurses perceived an inability of students to reflect on their learning, causing a perceived gap between theory and practice: *You can't blame them, they have never been taught to reflect upon what they do [...] Sometimes they go like sheep [...] in one direction and don't realize that the reality is much richer and more complex. We take care of people [...]*(N13).

### Building bridges between clinical settings and the university

The need to establish a common ground and connection between the university and hospital clinical settings was emphasized. Nurses spoke of how the university guides students' training, however, the clinical reality dictates that the training program is implemented at the hospital, and sometimes students are poorly prepared: *They should undertake more clinical practice sessions. It is true that, some time ago, there was little theory and lots of practice, but now they don't touch the patients, nor live in proximity with them, they just want to look at them from a "window". I think there is too much theoretical content and little reality.*(N16)

Nurses felt that the training program should also be designed by the clinical settings themselves. This would facilitate students' contact with the actual health care setting: *Being able to organize their reception from the hospital makes it easier to organize and adapt to our working needs, while not being imposed external conditions by the Nursing School, which doesn't know the clinical setting.*(N10)

On the other hand, clinical practice was considered as the real testing ground of students' knowledge, skills and abilities. Clinical practice was seen as the last step before becoming a professional. 

Nurses reported how the monitoring of students is generally carried out by nurse managers, who have no close contact with students during their practice period. Another aspect considered was the system for selecting the associate professor in charge of monitoring clinical practice. Some accounts described how this system (established by the Nursing School) disappointed many nurses, resulting in fewer nurses involved in students' training: *It seems like the university goes its separate way. There are nurses who have involved themselves in students' training, outside their working hours. What did the university do? They selected other less involved people instead and did not consider the [nurses'] dedication and commitment.*(N15)

## Discussion

In our study, nurses emphasized the key importance of the first contact with students. Previous studies^1^ ^,^ ^4^ ^-^ ^6^, reported that the way students are welcomed on the first day makes them feel accepted and motivated to learn. 

The attitude and motivation of students were positively evaluated by the nurses in our study during clinical practice training. Previous authors^3^ similarly found that there are students who are eager to learn while others have little interest. However, the reason for this passive attitude might be influenced by nurses' attitudes. In this way, previous studies^1^ ^,^ ^4^ ^-^ ^5^ ^,^ ^9^ ^-^ ^10^ ^,^ ^18^ described how nurses' attitudes affect students' eagerness to learn, and how students were more active when they felt supported by their nurse in the clinical practice setting. Nurses play an important role in enhancing students' clinical self-efficacy. Students gain many skills by watching what their peers do, and this helps them to gain self-efficacy and to achieve clinical self-esteem^4^.

Consistent with our results, previous studies^19^ have described the rejection of students by nurses during clinical practice. We found that some nurses avoid having students as this increases their workload and can be problematic. The acceptance and informal socialization of students as an important mechanism for facilitating integration, the knowledge of the ward culture and the encouragement of student learning and motivation^19^. In our study, nurses did not reveal their role in learning, leaving all learning responsibility to nursing students. Plus, the role of the nurse was less active than described in previous studies^1^ ^,^ ^3^ ^-^ ^6^. 

Nurses in this study stated that their length of experience may be a hindrance towards nursing students' training. Heavy workloads, workforce shortages in hospitals, busy wards, an overload of students and their being treated as workers are all obstacles to students' training during clinical practice as well as factors influencing nurses' teaching abilities^20^. Despite this, nurses expressed a high satisfaction in relation to mentoring students, but admitted that this also necessitated extra work^10^.

Previous studies have concentrated on the qualities of a good mentor; student support, motivation and involvement during practice^2^ ^,^ ^5^ ^-^ ^6^. However, little has been said about the nurse who is not willing to have any nursing students under their care. In our study, two related features have been identified; negative experiences with the Nursing School and the absence of reward. This supports previous research^10^ which stated that previous negative experiences with students (related to nurses' feeling unprepared for teaching and having to deal with time limitations ) influences nurses. Such nurses tend to focus on their own needs and view students as an imposition. 

In order to define the student's role in clinical practice, first of all, the role of the nurse must be defined. A range of aspects covered by nurses providing training include: the objectives of hospital nursing, the holistic perspective of nursing, professional development for their role as a RN, the rules of the ward, clinical procedures and how to take care of patients^4^ ^-^ ^6^ ^,^ ^9^ ^,^ ^11^ ^,^ ^20^ ^-^ ^21^. In this sense, nurses consider their responsibility to explain to students the priorities in clinical settings. Also, nurses are responsible for the training of nursing students both for their future role as competent nurses and for their capability of working as colleagues^5^ ^-^ ^6^ ^,^ ^10^. These aspects were not identified in our study. 

The findings of this study confirm a reported tendency for using technology, to the detriment of direct patient contact. Millennial students, those born between 1980 y 2000, tend to learn through trial and error within active learning environments^22^. Furthermore, the importance of flexibility when scheduling clinical practice is highlighted by this study, together with the integration of technology into the learning process. These learning requirements may clash with clinical reality, however they can be implemented by using modern information and communication technologies^22^. 

Our results show a lack of reflexivity on the part of students. The reflection process enhances autonomy, encourages personal growth, helps integrate theory and practice and develops a greater clinical experience^21^ ^-^ ^23^. Nurses are essential for the development of this reflection process and the acquisition of clinical experience^21^ ^-^ ^23^. When RNs and students are encouraged to participate in reflection processes, this facilitates the integration of different ways of thinking when dealing with clinical situations^21^ ^,^ ^23^.

The integration between theory and practice is more effective when strong links exist between the college and the clinical staff^24^. Consistent with our results, previous studies^1^ found that staff nurses felt that nursing students were not trained for the actual care goals. These authors pointed out that more than half of the nurses studied believed that Nursing Schools were not providing a quality education, and that nursing students are unprepared. Not withstanding the fact that nurses thought the theoretical knowledge of students was of a high standard, they questioned their ability to apply it to real situations. Nurses consider their training to be far stricter than current study programs, as, in the past, students were part of the ward workforce^1^. Our study supports these findings, as the view of nurses was that, at the time, they had greater hands-on experience. A possible explanation for this difficulty to integrate theory and practice could be due to the fact that previous studies have reported the existence of a hidden curriculum within nursing studies^25^. A hidden curriculum generally aims towards the unplanned transmission of values and behaviours, as opposed to the planned teaching of knowledge and skills. It is conformed by the implicit values held by the nurses, and can be transmitted through both verbal and nonverbal messages. Thus, learning through this kind of curriculum is a common experience among students in clinical settings^5^ ^,^ ^25^. Some aspects of this hidden curriculum are the nurse's behaviour, the nurse's perception as a reference model for students, and the clinical placements within which the clinical knowledge and skills are applied in the context of real patients, real diseases, real resources, and real social limitations^5^ ^,^ ^25^.

Monitoring students during clinical practice requires a person in charge to carry out a continuous assessment of their learning. In a previous study^1^ which described nurses' experience related nursing student, 47.71% of the nurses interviewed agreed with the statement: "I would not have to spend extra time with nursing students if the instructors supervised them". Furthermore, 45.72% of the staff nurses surveyed thought they should not teach students when clinical nursing instructors were being paid for that. Our results show that the selection process for associate professors is carried out without regard to the RN. Previous studies^19^ ^,^ ^20^ found that the candidates' teaching ability through reflective teaching and learning should be considered when recruiting associate professors. In Spain, and consistent with our results, the associate professors of Nursing Schools are selected without regard to the practice settings and their RN^19^. Therefore, a recommended strategy for favouring the implication of RNs in the learning and follow-up of their nursing students would be to recruit RNs directly from clinical settings.

This is a qualitative research study conducted within a specific context, that of a country in southern Europe. However, to counter this, similar qualitative studies should be carried out in multiple environments in order to gain a better understanding of the phenomenon. 

## Conclusions

The attitude and involvement of the RN can influence students' attitudes. It is necessary to define the role of the nurse in order to develop clear models for students to follow and define their own role in clinical practice. There must be a common line of work between the university and the clinical settings in order to facilitate student learning. 

The involvement of RNs in the training of nursing students should be considered a priority for nursing schools and managers of clinical centres, due to the fact that RNs represent the crucial communication link between the academic world and the clinical sites. Registered nurses play an essential role in teaching students important values and in guiding students through the role of nurses in clinical care contexts.

The results of this study can be used by university nursing departments to improve training curricula and clinical practice. A proper understanding of nurse mentors' perceptions during practice can help improve the quality of learning and develop more realistic study plans together with those responsible within the clinical placements.
